# Secondary School Pupils’ Mental Wellbeing Is Associated with Belonging to a Perceived Minority and Experiencing Discrimination

**DOI:** 10.3390/children8020071

**Published:** 2021-01-21

**Authors:** Pinja Kokkonen, Christina Athanasopoulou, Helena Leino-Kilpi, Evanthia Sakellari

**Affiliations:** 1 Department of Nursing Science, University of Turku, 20014 Turku, Finland; pinja.p.kokkonen@gmail.com (P.K.); chatha@utu.fi (C.A.); helena.leino-kilpi@utu.fi (H.L.-K.); 2 Turku University Hospital, 20521 Turku, Finland; 3 Department of Public and Community Health, University of West Attica, 11521 Athens, Greece

**Keywords:** schools, wellbeing, adolescents, school-based health promotion, mental health

## Abstract

Adolescents’ mental health is a global issue and there is a growing interest in tackling mental health in schools. The study aimed to assess secondary school pupils’ mental wellbeing and the factors related to their mental wellbeing (sociodemographic characteristics, perception of belonging to a minority, and discrimination). Data were collected from 12–17-year-old pupils of a Finnish secondary school via an online questionnaire. Data were analyzed with descriptive statistics and ANOVA for group comparisons. Participants’ (Ν = 114) mental wellbeing scores were above average (47.5, max. 70). Worse family relations were independently associated with worse mental wellbeing. Additionally, significantly lower scores on mental wellbeing were found among pupils who thought they belonged to a minority due to appearance, sexual orientation, and/or chronic disease. Participants who had experienced discrimination had significantly lower mental wellbeing scores in comparison to those who never had such an experience. In conclusion, mental health promotion interventions which promote good family relations should be targeting different youth groups in order to address their specific needs. Thus, screening programs which identify pupils who are at risk or belong to minority groups are needed, in order to direct them to proper services when needed and/or implement mental health promotion interventions accordingly.

## 1. Introduction

Research shows that mental wellbeing declines as young people move through adolescence, while it is also shown that adolescents with good emotional and physical health tend to cope with the challenges of adolescence and have an easier transition into adulthood [1]. Adolescents’ mental health is a global issue and there is a growing interest in tackling mental health in schools [2]. In order to implement successful interventions, adolescents are important to consider [3], as well as the factors affecting their mental wellbeing.

There is some knowledge concerning the factors associated with mental wellbeing among different age groups. The growing environment [4] and social support in general [5] are related to both adults’ and children’s wellbeing. Social support is defined as the instrumental and/or expressive provisions, real or perceived, given by the community, social networks, and intimate relationships [6]. The positive role of social support in wellbeing is apparent in various studies with adolescents [7,8]. On the other hand, children’s low levels of wellbeing are associated with living with only one parent or having conflicts in the family [5,9] which has shown the strongest association with child unhappiness [10].

Parental socioeconomic status (employment, income, educational status) has a high impact on the child’s mental health outcomes [11]. However, studies have shown contradictory findings about the effects of finances. One showed that belonging to the highest income group was related to lower levels of wellbeing among 11 year olds [9], while another study showed that having less money than friends was related to lower levels of wellbeing [5]. Similarly, it has recently been found that socioeconomic status was not significantly related to the children’s psychological wellbeing, with parent involvement and the parent–child relationship playing a complete mediating role [12]. Parents’ employment status affects children’s wellbeing in terms of the work impairing the developing bond between themselves and young children, especially when they work long hours or evening and night shifts. In addition, the stress that they bring home from their jobs can detract from their parenting skills, undermine the positive atmosphere at home, and, finally, introduce stress into their children’s lives [13].

Furthermore, schoolwork has been an additional factor for adolescents’ wellbeing, with a high proportion of 11-, 13-, and 15-year-old of pupils (35%) reporting that they feel some or a lot of pressure from schoolwork [14], which has been associated with depressive symptoms [15], more frequent subjective health complaints, and lower levels of life satisfaction [14].

Based on a meta-analysis of 134 studies conducted in 2009 [16], it appears that another factor, perceived discrimination, has a significant negative effect on both mental and physical health. Discrimination is defined as the unfair treatment of a person or group due to particular characteristics, such as gender, age, ethnicity, sexual orientation, or disability [17], while perceived discrimination exists when people themselves perceive or experience discrimination [18]. In fact, a recent study reported that perceived discrimination was associated with 1.86 higher odds of mental health problems [19].

In addition, evidence suggests that there is an association between adolescents’ mental health problems and belonging to some minority, for example, young people belonging to a sexual minority is associated with higher suicide ideation compared to peers [20]. Similarly, there is increased risk of suicide for adolescents belonging to indigenous ethnic minorities [21]. In fact, a recent review notices minority status as an influencing factor for children’s and adolescents’ mental health when they migrate to Europe [22]. Therefore, belonging to a minority is often related to mental health problems. However, factors related to mental health problems and mental wellbeing are somewhat different [9].

While several studies explore the factors of wellbeing among adolescents [14,23,24,25], to our knowledge, the literature does not address the consequences of low levels of mental wellbeing. However, the literature addresses the effects of mental health problems in adolescents. For example, as stated by the WHO [14], mental health problems can have damaging effects on young people’s social, intellectual, and emotional development and, consequently, on their future. In addition, it is stated that adolescent mental health problems are risk factors for future mental distress and psychopathology [26].

According to the WHO [27], adolescents should receive adequate attention in all policies, strategies, and programs that are relevant to them and they should be heard in order to ensure that all initiatives meet their needs. Hence, this study contributes by focusing on adolescents’ mental wellbeing and the factors that are associated with mental wellbeing in order to provide implications for practice within mental health promotion in the school setting. The study’s research questions were:

1. What is the adolescents’ level of mental wellbeing?
2. Are socioeconomic factors associated with the adolescents’ level of mental wellbeing?
3. Is the perception of belonging in a minority a factor associated with the adolescents’ level of mental wellbeing?
4. Is the perception of experiencing discrimination a factor associated with the adolescents’ level of mental wellbeing?

## 2. Materials and Methods

### 2.1. Participants and Setting

A descriptive survey design was used. Data collection was conducted during school hours in one randomly selected public secondary school in Western Finland. School education in Finland is based on the national core curriculum for basic education (integrated primary and lower secondary education), which is developed by the Finnish National Agency for Education, while the local education authorities and the schools themselves draw up their own curricula within the framework of the national core curriculum [28]. The selected school did not implement any specific health promotion program at the time of data collection. Pupils’ local schools are determined by their address of residence [29] and the selected school is located in the city of Turku and does not belong in any exceptional socioeconomic community.

All pupils (Ν = 225) in the school were recruited for the study with the support of the school principal and teachers. The inclusion criteria were: ability to understand and use the Finnish language, parent’s permission to participate, and the adolescent’s own willingness to participate. A total of 114 pupils participated in the study (response rate 50.7%). Participants were pupils aged 12–17 years (mean age 14.2, SD = 1.04). Most participants were 16 years old or above, female, and lived with both parents (Table 1). A few participants had been diagnosed with emotional disorders (*n* = 10, 8.8%), about a fifth (*n* = 24, 21.1%) perceived they belonged to a minority, and a bit less than half of them (*n* = 52, 45.6%) experienced discrimination (40.4% “sometimes”, 5.3% “often”) (Table 1). The mean score of adolescents’ mental wellbeing was 47.5 (SD = 13.1) out of maximum of 70.

### 2.2. Data Collection and Measures

Data were collected by an online questionnaire, which included the following: (1) background information; (2) Warwick–Edinburgh Mental Well-being Scale (WEMWBS); (3) perceptions of belonging to a minority and experiencing discrimination.

First, background information was collected, including: age (Date of Birth), gender (participant could fill the box), living situation (Both parents; One parent; On my own; Living with relatives other than parents; Foster care; Group home; Psychiatric hospital; Homeless; Other, please describe), educational level of the father (Basic education; Tertiary education; Don’t know/Don’t want to answer), educational level of the mother (Basic education; Tertiary education; Don’t know/Don’t want to answer), father’s occupation status (Does your father work?: Yes; No; I don’t know; I do not have a father/I do not meet him), mother’s occupation status (Does your mother work?: Yes; No; I don’t know; I do not have a mother/I do not meet her), diagnosed emotional difficulties (Have you been given a name or a diagnosis for your emotional difficulties? If yes please specify), relation with family (on a 10-point scale from Very bad to Excellent), pressure from homework (How much pressure you feel from homework?: None; Little; Enough; Very much).

Second, mental wellbeing was measured with the Finnish version [30] of the WEMWBS [31]. The WEMWBS is a 14-item scale addressing aspects of positive mental health, including psychological, eudemonic, and hedonic perspectives. Items are scored on a Likert scale, ranging from 1 = “never” to 5 = “all the time”. The scale is scored by summing responses to each item. All questions are equally weighted. Scores can range from a minimum of 14 to a maximum of 70 points. The WEMWBS’s psychometric properties have been shown to be good. In this study, Cronbach’s alpha (0.96) showed high internal consistency.

Third, perceptions of belonging to some minority were measured by using questions from the Finnish Youth Barometer study [32]: “Do you feel that you belong to a minority in the following matters?” with the options of “ideological conviction or opinion”, “religious or non-religious conviction”, “ethnic background”, “appearance (such as skin colour, dress...)”, “sexual orientation (such as gay...)”, “disability or chronic illness”, “gender identity (such as transsexual)”, and “other minority” and the question “Have you experienced discrimination?” with the options “yes often”, “yes sometimes”, “no”, and “can’t say”.

### 2.3. Data Analysis

Quantitative variables were expressed as mean values or as median values (interquartile range = IQR). Qualitative variables are expressed as absolute and relative frequencies. The Kolmogorov–Smirnov test was used to test if the normality assumption was satisfied and no deviation from normality was found. Analysis of variance (ANOVA) was used for the comparison of mental wellbeing score among three or more groups and Bonferroni correction was used in order to control for type I errors in multiple comparisons. For the comparison of means between two independent groups, a Student’s *t*-test was used. Spearman correlation coefficient was used to explore the association of mental wellbeing score with the score that reflected the quality of the family relationships. Multiple linear regression analysis in a stepwise method (*p* for removal was set at 0.1 and *p* for entry was set at 0.05) was used in order to identify independently associated parameters with the mental wellbeing score. Adjusted regression coefficients (β) with standard errors (SE) were computed from the results of the linear regression analyses. Additionally, standardized regression coefficients were performed as a measure of the effect of independent variables. All *p* values reported are two-tailed. Statistical significance was set at 0.05 and analyses were conducted using SPSS statistical software (version 22.0).

### 2.4. Ethical Considerations

The study has ethical approval from the Ethical Committee of Turku University (Number 29/2016). Since data collection took place during school time, the national Finnish guidelines were followed and hence the school principal gave permission to conduct the study [33]. Potential participants and their parents/guardians were informed about the study with a separate letter in which anonymity and voluntariness were emphasized. Pupils’ response to the online questionnaire was considered as their informed consent. Parents/guardians had the possibility to deny their child’s participation. Moreover, although pupils had permission from their parents to participate, the participation was still their own choice. Last, permission to use the instruments was obtained from the authors who developed them.

## 3. Results

In terms of sociodemographic variables, higher mean scores of mental wellbeing were found among female adolescents, aged more than 16 years, whose mothers worked, whose father’s educational level was ≤12 years and mother’s educational level was more than 12 years, and without or with little pressure from homework (Table 2). Lower mean scores of mental wellbeing were found in adolescents living with one parent and rating pressure from homework as “Enough” or “Very much” (Table 2). A statistically significant and positive correlation of mental wellbeing was found among those with good family relations (r = 0.37, *p* < 0.001).

The association of variables concerning minorities and discrimination with mental wellbeing is presented in Table 3. Statistically significantly lower scores on the mental wellbeing dimension were found in cases where adolescents perceived that they belonged in a minority due to appearance, sexual orientation, or chronic disease. Furthermore, participants who had experienced discrimination had significantly lower scores on mental wellbeing in comparison to those who never had such an experience.

A multiple regression analysis was conducted with mental wellbeing as the dependent variable (Table 4) and it was found that adolescents with more educated mothers scored higher in the mental wellbeing dimension. Lower mental wellbeing was associated with worse relationships in the family, a feeling of belonging to a minority due to sexual orientation or chronic disease, and discrimination experience. Overall, relation with family and discrimination, followed by belonging to a minority due to sexual orientation, had the greatest effect on mental wellbeing, as indicated by the standardized regression coefficients of the model (coefficient b).

## 4. Discussion

This study aimed to describe secondary school pupils’ mental wellbeing and to explore the relation of their mental wellbeing with sociodemographic characteristics, belonging in a perceived minority, and experiencing discrimination.

In this study, the mean score of adolescents’ mental wellbeing was 47.5 out of a maximum of 70, close to earlier results found ten years ago among English and Scottish secondary school pupils [34] with a mean WEMWBS score of 48.8 (SD = 8.6). With regard to the socioeconomic characteristics, in the current study, higher mean scores of mental wellbeing were found among participants whose mothers worked, whose father’s educational level was 12 years or less, and whose mother’s educational level was more than 12 years. These findings are partly supported by the literature that shows that adolescents with a low socioeconomic status are exposed to a higher risk of developing mental health problems [35]. In addition, it has been found that among those adolescents who are socioeconomically disadvantaged, self-reported depression has been increasing through the years [36].

With regard to parents’ educational level, the current study reports the interesting finding that participants had higher mental wellbeing scores when their father had basic or lower education and their mother’s educational level was high, while previous studies report only about the similar educational level of both parents in relation to their children’s wellbeing, with no conclusive results. More specifically, an earlier study [37] reported that adolescents whose parents have higher education do not score higher on psychological wellbeing. Similarly, a recent study [38] reported that parents’ higher educational level was related to adolescents’ lower mental wellbeing. On the other hand, another study [39] demonstrated that relationships between emotional, behavioral, social, and school wellbeing are generally similar for adolescents regardless of their parents’ educational level. As for homework pressure, it seemed to be related to lower mean scores of mental wellbeing. Similarly, another recent study found that depression scores have been found to be higher in adolescents who spent long hours on homework/studying [40].

The literature indicates that parental support and strong family bonds are linked to positive emotional wellbeing in adolescence [41]. The results of this study highlighted the meaning of family relations for the pupils’ mental wellbeing. Excellent relationships in the family were found to be independently associated with high mental wellbeing. In recent studies, having good family relationships was included in children’s own perception of their wellbeing and adolescents have described mental health as being loved or loving others [42]. The results of our study are in line with earlier findings [43], where family members, and time spent with them, have had essential roles in adolescents’ wellbeing. Furthermore, adolescents with positive mental health report high family support more often than others and high parental involvement is associated with adolescents’ decreased likelihood of having poor mental health [44]. In addition, youth who felt more connected to parents reported lower levels of depressive symptoms, suicidal ideation, non-suicidal self-injury, and conduct problems [45].

Furthermore, the parental role is obvious, for example, there is strong evidence of the meaning of parenting intervention in children’s behavioral problems [46]. Even though the literature supports that the impact of families on adolescents’ mental wellbeing and parenting interventions are quite cost-effective to address children’s mental health promotion [47], there are, however, opposite findings too [48]. Interestingly, a recent review shows that mental health promotion interventions implemented for non-clinical samples of Finnish school-aged adolescents do not include parents in their target group, but are mostly targeted to the individuals [49]. Thus, given the fact that families are positive factors contributing to adolescents’ mental wellbeing, health professionals should take that under consideration and refocus their interventions that aim to improve adolescents’ mental wellbeing. Well-planned interventions can support adolescent–parent relationships. Although adolescents get more independent as they grow older, they need support from their parents, who should be included in these interventions when planning mental wellbeing interventions.

Adolescents experiencing discrimination exhibited lower scores in mental wellbeing. As the study sample was small, it may be relevant to explore the percentages compared to larger samples. In our study, 45.6% of the participants reported that they have experienced discrimination. This is similar to an earlier study in a larger sample among Finnish young people that reported 55% [32]. However, 26% of secondary school pupils reported discriminatory bullying, which is not totally comparable concept since, as stated in the literature [50], it is as an act of adolescent arrogance that aims at discriminating, and a form of abuse and victimization, while the term discrimination refers to a behavior that directly or indirectly distinguishes, excludes, restricts, or prefers a person. Similar to our findings, positive wellbeing was recognized to have a reverse correlation with adolescents’ race/ethnic discrimination [51]. Although high levels of mental health problems and low levels of mental wellbeing should not be equated directly, experience of discrimination also has an increasing effect on mental health problems. There is a negative association between perceived societal and interpersonal discrimination and mental health [52], and high racial discrimination associated with greater odds of psychological stress and stress related to suicidal thoughts [53]. Race/ethnic discrimination is also attached to depressive and internal and external symptoms [51]. In addition, it has been found that perceived racial discrimination during adolescence has significant harmful effects decades later on black men’s mental health [54].

Although mental wellbeing was lower in all groups of pupils who identify as belonging to some minority, the difference was not statistically significant in all of the groups. Due to the few respondents in minority groups (*n* = 3–7), the results concerning the specific minority groups are tentative. More specifically, belonging to a sexual minority (*n* = 7) was found to be associated with a lower score on mental wellbeing. There is previous evidence showing that belonging to a sexual minority is related to problems with adolescents’ mental health [20,53]. It was found that sexual minority adolescents in the UK, who experience disparities in mental health, were more likely to experience high depressive symptoms and self-harm [55], similar to the results of an earlier study among adolescents from the Canadian province of British Columbia [56]. Similarly, in another study, it was found that sexual minority youth had reported significantly higher rates of suicidality and depression symptoms in comparison to heterosexual youth [57], with a recent study reporting the same results [58].

Belonging to a minority due to chronic disease was also found to be associated with a lower score on mental wellbeing in our study, with four participants reporting to have a chronic disease. Different studies have concluded that many children and adolescents with chronic health conditions experience mental health problems [59]. However, when focusing on mental wellbeing again, youth with neuromuscular disorders, diabetes, or cerebral palsy scored higher levels of mental wellbeing with the WEMWBS compared to the general population [60]. Thus, it can be seen that the two-fold support from the literature supports that it is integral to notice the meaning of measuring mental wellbeing along with mental health problems. Although belonging to a minority seems to be related to mental health problems and, in our study, to low mental wellbeing, it is also possible that belonging to a minority can strengthen young people.

It should be pointed out that discrimination on the basis of religion, ethnic background, appearance, sexual orientation, disability, gender identity, or being in any minority is a global phenomenon [61]. In the European Union, more than half say that there is widespread discrimination in their country and, more specifically, 53% say that this is on the basis of sexual orientation and 59% on the basis of ethnic origin [62]. Due to discrimination, adolescents around the globe can face long-term academic, psychological, and social consequences. How adolescents are impacted by this discrimination depends on their cognitive ability to perceive the bias, the context in which the bias occurs, and the resources they have to help cope with the bias and, of course, on culture [61]. Additionally, parental responses to, for instance, a youth’s disclosure of sexual minority orientation may also differ based on race/ethnicity or cultural levels of acceptance of sexual minority individuals [63].

In this study, there are some limitations to be considered. Firstly, the results of the study cannot be generalized since it took place in one city in Finland, and caution is needed when interpreting the results. Secondly, the number of respondents in minority subgroups was very small (*n* = 3–7). Thirdly, data were collected by a self-reported questionnaire which may cause responses to be biased. On the other hand, all the instruments used are validated and, moreover, self-reported questionnaires offer privacy to the participants in order for them to fill the answers honestly. Finally, collecting data online was an advantage for the adolescents since they are very familiar with new technologies and the internet. All pupils aged 12–17 years were invited to take part in the study, while the response rate was at an acceptable rate (50.7) [64].

## 5. Conclusions

When planning mental health promotion activities, pupils should be included as active participants and their perspectives need to be acknowledged. The results of this study suggest implications for professionals who design and implement young people’s mental health promotion programs. As shown in this study, belonging to a perceived minority has an impact on mental wellbeing. Hence, specific mental health promotion interventions should be adapted in different youth groups in order to address their specific needs. Additionally, it is essential to acknowledge that challenging situations do not always lead only to problems, but may turn into strengthening resources and lead to empowerment.

Furthermore, since the well-known importance of family relations has been highlighted, interventions promoting good family relations will have a positive impact on pupils’ mental wellbeing. Thus, parents should be included in supporting their children’s mental wellbeing. Finally, health professionals who work with young people should implement screening programs in order to identify pupils at risk and direct them to proper services when it is needed.

Systematic research using a valid mental wellbeing instrument will also be important in the future, as the research of mental wellbeing is now fragmented and scarce. Further research is needed in order to study mental wellbeing among pupils with perceived minority experience. Finally, further research may focus on mental health promotion interventions targeting specific young population groups in order to provide evidence on what may lead to positive outcomes for their mental health.

## Figures and Tables

**Table 1:** Participants’ characteristics (Ν = 114).

Characteristics	*n* (%)
Age (years)	
<16	48 (42.1)
≥16	66 (57.9)
Gender	
Male	49 (42.1)
Female	63 (55.3)
Other	2 (1.7)
Father’s occupation status	
Other *	24 (21.1)
Employed	90 (78.9)
Mother’s occupation status	
Other *	20 (17.5)
Employed	94 (82.5)
Educational level of the father	
Don’t know	47 (41.2)
≤12 years	27 (23.7)
>12 years	40 (35.1)
Educational level of the mother	
Don’t know	41 (36)
≤12 years	20 (17.5)
>12 years	53 (46.5)
Living with	
Both parents	72 (63.2)
One parent	34 (29.8)
Other **	8 (7)
Relation with family (1 = excellent to 10 = very bad), median (IQR)	2 (1–3)
Diagnosed emotional difficulties	
No	104 (91.2)
Yes	10 (8.8)
Pressure from homework	
None/little	59 (51.8)
Enough/very much	55 (48.2)
Belonging to a minority due to:	
Ideology	
No	107 (93.9)
Yes	7 (6.1)
Religion	
No	107 (93.9)
Yes	7 (6.1)
Origin	
No	110 (96.5)
Yes	4 (3.5)
Appearance	
No	107 (93.9)
Yes	7 (6.1)
Sexual orientation	
No	107 (93.9)
Yes	7 (6.1)
Chronic disease	
No	110 (96.5)
Yes	4 (3.5)
Gender identity	
No	110 (96.5)
Yes	4 (3.5)
Other	
No	111 (97.4)
Yes	3 (2.6)
Belong to any minority	
No	90 (78.9)
Yes	24 (21.1)
Have you ever experienced discrimination	
No	47 (41.2)
Can’t answer	15 (13.2)
Yes	52 (45.6)

* Other: I don’t know; I don’t have a mother/father; I do not meet him/her. ** On my own; Living with relatives other than parents; Foster care; Group home; Psychiatric hospital; Homeless; Other, please describe.

**Table 2:** Associations of sociodemographic variables with the level of mental wellbeing.

	Warwick–Edinburgh Mental Well-Being Scale Score		
	Mean	SD	*p*
Age (years)			
<16	46.8	14.3	0.620 ǁ
≥16	48.0	12.3	
Gender			
Male	47.2	16.1	0.733 ǁ
Female	48.1	10.6	
Father’s occupation status			
Other *	46.5	13.9	0.673 ǁ
Employed	47.8	13.0	
Mother’s occupation status			
Other *	44.7	12.1	0.285 ǁ
Employed	48.1	13.3	
Educational level of the father			
Don’t know	43.6	14.7	0.053 ¶
≤12 years	50.6	11.6	
>12 years	50.0	11.0	
Educational level of the mother			
Don’t know ^A^	42.3 ^C^	15.2	0.004 ¶
≤12 years ^B^	48.4	12.2	
>12 years ^C^	51.2 ^A^	10.2	
Living with			
Both parents ^A^	48.9 ^B^	12.9	0.018 ¶
One parent ^B^	42.7 ^A,C^	12.9	
Other ^C^	54.9	10.4	
Diagnosed emotional difficulties			
No	47.0	13.0	0.209 ǁ
Yes	52.5	13.6	
Pressure from homework			
None/little	50.6	11.5	0.008 ǁ
Enough/very much	44.1	14.0	

ǁ Student’s *t*-test; ¶ ANOVA; ^A^, ^B^, ^C^ indicate significant differences between groups after Bonferroni correction * Other: I don’t know; I don’t have a mother/father; I do not meet him/her.

**Table 3:** Associations concerning minorities and discrimination with mental wellbeing.

	Warwick–Edinburgh Mental Well-Being Scale Score		
	Mean	SD	*p*
Belonging to a minority due to:			
Ideology			
No (*n* = 107)	47.7	13.3	0.467 ǁ
Yes (*n* = 7)	44.0	10.1	
Religion			
No (*n* = 107)	48.0	12.8	0.158 ǁ
Yes (*n* = 7)	40.7	16.9	
Origin			
No (*n* = 110)	47.6	12.8	0.846 ǁ
Yes (*n* = 4)	46.3	23.0	
Appearance			
No (*n* = 107)	48.2	12.4	0.020 ǁ
Yes (*n* = 4)	36.4	19.2	
Sexual orientation			
No (*n* = 107)	48.2	12.9	0.035 ǁ
Yes (*n* = 7)	37.4	13.3	
Chronic disease			
No (*n* = 110)	48.0	13.0	0.039 ǁ
Yes (*n* = 4)	34.3	11.6	
Gender identity			
No (*n* = 110)	47.9	12.9	0.120 ǁ
Yes (*n* = 4)	37.5	17.6	
Other			
No (*n* = 111)	47.8	12.9	0.221 ǁ
Yes (*n* = 3)	38.3	21.8	
Belong to any minority			
No (*n* = 90)	48.4	12.6	0.141 ǁ
Yes (*n* = 24)	44.0	14.6	
Experienced discrimination			
No ^A^ (*n* = 47)	52.1 ^C^	12.4	0.006 ¶
Can’t answer ^B^ (*n* = 15)	45.1	9.6	
Yes ^C^ (*n* = 52)	44.1 ^A^	13.5	

ǁ Student’s *t*-test; ¶ ANOVA; ^A^, ^B^, ^C^ indicate significant differences between groups after Bonferroni correction.

**Table 4:** Multiple linear regression analysis for mental wellbeing dimensions.

	β ⱡ	SE ǁ	b ¶	*p*
Mother’s educational level:				
Other, reference				
>12 years	4.83	2.19	0.18	0.029
Relation with family (1 = excellent to 10 = very bad)	−2.00	0.59	−0.29	0.001
Belonging to a minority due to sexual orientation:				
No, reference				
Yes	−10.13	3.99	−0.22	0.013
Belonging to a minority due to chronic disease:				
No, reference				
Yes	−12.23	5.89	−0.17	0.040
Have you ever experienced discrimination:				
No, reference				
Can’t answer	−2.52	3.51	−0.07	0.474
Yes	−6.92	2.44	−0.26	0.006

ⱡ Regressions coefficients; ǁ standard error; ¶ standardized regression coefficient.

## Data Availability

Data sharing not applicable due to privacy and ethical restrictions.
